# Long-term Trends in Incidence and Mortality of Intrahepatic and Extrahepatic Bile Duct Cancer in Japan

**DOI:** 10.2188/jea.JE20130122

**Published:** 2014-05-05

**Authors:** Mai Utada, Yuko Ohno, Tomoko Tamaki, Tomotaka Sobue, Ginji Endo

**Affiliations:** 1 Department of Mathematical Health Science, Graduate School of Medicine, Osaka University, Suita, Osaka, Japan; 1 大阪大学大学院医学系研究科数理保健学; 2 Department of Social and Environmental Medicine, Graduate School of Medicine, Osaka University, Suita, Osaka, Japan; 2 大阪大学大学院医学系研究科社会環境医学; 3 Department of Preventive Medicine and Environmental Health, Graduate School of Medicine, Osaka City University, Osaka, Japan; 3 大阪市立大学大学院医学研究科産業医学

**Keywords:** intrahepatic bile duct cancer, extrahepatic bile duct cancer, incidence, mortality

## Abstract

**Background:**

A report of multiple cases of bile duct cancer at a Japanese printing company raised concern about such cancers. We examined long-term trends in bile duct cancer in Japan.

**Methods:**

Data from 4 population-based cancer registries were used to calculate incidence between 1985 and 2007, and vital statistics were used to estimate mortality between 1985 and 2011. Age-standardized rates were calculated and analyzed using a joinpoint regression model.

**Results:**

Among men, the incidence rate of intrahepatic bile duct cancer increased throughout the observation period; among women, it increased until 1996–1998 and remained stable thereafter. The incidence rate of extrahepatic bile duct cancer was stable in men and decreased from 1993–1995 in women. In people aged 30 to 49 years, the incidence rates of intra- and extrahepatic bile duct cancer remained stable or decreased. The mortality rate of intrahepatic bile duct cancer increased in both sexes and in all age groups since 1996, while that of extrahepatic bile duct cancer decreased since 1992. In people aged 30 to 49 years, the mortality rates of intra- and extrahepatic bile duct cancer remained stable and decreased, respectively.

**Conclusions:**

The incidence and mortality rates of intrahepatic bile duct cancer remained stable or increased throughout the observation period. The incidence rate of extrahepatic bile duct cancer remained stable or decreased, and the mortality rate decreased since 1992. In people aged 30 to 49 years, the incidence and mortality rates of intra- and extrahepatic bile cancer remained stable or decreased.

## Abstract (ja)

**【背景】:**

印刷会社における胆管がんの多発が報告され、関心が高まっている。本研究では、日本における胆管がんの長期トレンドを検討した。

**【方法】:**

がん罹患は1985–2007年の4つの地域がん登録、がん死亡は1985–2011年の人口動態統計を用いた。年齢調整率はJoinpoint回帰モデルを用いて解析した。

**【結果】:**

全年齢の肝内胆管がんの罹患率は、男性では観察期間中増加し、女性では1996–1998年まで増加し、それ以降は一定であった。肝外胆管がんの罹患率は、男性では一定で、女性では1993–1995年から減少した。30–49歳の肝内胆管と肝外胆管の罹患率は、一定または減少した。男女共に肝内胆管がんの死亡率は1996年から増加し、肝外胆管の死亡率は1992年から減少した。30–49歳の肝内胆管の死亡率は一定で、肝外胆管の死亡率は減少した。

**【結論】:**

肝内胆管がんの罹患率と死亡率は、観察期間中一定か増加していた。一方、肝外胆管がんの罹患率は一定か減少で、死亡率は1992年から減少していた。30–49歳では、肝内胆管と肝外胆管の罹患率と死亡率は一定か減少していた。

## INTRODUCTION

In 2012, Kumagai et al reported that a high percentage of workers at a printing company in Osaka had developed and died from intrahepatic bile duct (IHBD) or extrahepatic bile duct (EHBD) cancer.^1^ In March 2013, the Ministry of Health, Labour, and Welfare reported that, among 70 men who had worked in the offset color proof-printing section of the printing company, 16 men had developed IHBD or EHBD cancer and 7 had died from these cancers.^2^ Their ages at diagnosis and death were 25 to 45 years (mean, 36 years) and 27 to 46 years (mean, 37 years), respectively. The incidence and mortality rates of IHBD and EHBD in people younger than 50 years are extremely low in Japan.^3^ Thus, occupational chemical exposure was suspected to be the cause of the high incidence and mortality rates of IHBD and EHBD cancer.^1^

Trends in the incidence and mortality of IHBD and EHBD cancer must be monitored to determine their respective risks. However, in the International Classification of Disease (ICD), IHBD and EHBD cancer are categorized as “liver and intrahepatic bile ducts” and “gallbladder and extrahepatic bile ducts,” respectively. Therefore, individual trends in these cancers cannot be observed.

We investigated long-term trends in IHBD and EHBD cancer incidence and mortality by age group in Japan.

## METHODS

### Data sources

We selected 4 prefectures in Japan: Miyagi, Yamagata, Fukui, and Nagasaki. These 4 prefectures had population-based cancer registries with long-term (ie, >20 years), high-quality data. A previous study concluded that use of data from these 4 prefectures was a provisionally acceptable way to evaluate cancer incidence trends in Japan.^4^

We obtained cancer incidence data from each prefectural population-based cancer registry for the period from 1985 through 2007. Cancer mortality data were obtained from vital statistics from 1985 through 2011. Cancers were classified according to the International Classification of Diseases for Oncology (ICD-O) for incidence and according to the ICD for mortality. The classification of ICD-O for incidence was converted into ICD codes. We used the ICD Ninth Revision (ICD-9) for data until 1994 and the 10th revision (ICD-10) for data from 1995 or later. We analyzed IHBD cancer (ICD-9, 155.1; ICD-10, C22.1) and EHBD cancer (ICD-9, 156.1; ICD-10, C24.0).

### Statistical analysis

A previous study reported regional differences in IHBD and EHBD cancer mortality in Japan^5^^,^^6^; moreover, incidences of these cancers are believed to differ by region. Therefore, we employed a method used by the Monitoring of Cancer Incidence in Japan (MCIJ) Project^7^^–^^12^ to estimate nationwide cancer incidence in Japan. This estimation method uses the arithmetic means of incidence rates, by site, sex, and 5-year age group, in the selected registries. Estimated nationwide cancer mortality in Japan was calculated using the same method, and observed cancer mortality was obtained from the vital statistics of the selected prefectures. Correction coefficients (ie, the ratios of estimated to observed cancer mortality) were subsequently calculated by site and sex. To avoid bias due to prefectural differences in cancer incidence and mortality, and to obtain corrected estimates, the estimated uncorrected cancer incidences according to site, sex, and 5-year age group were multiplied by correction coefficients for each year. In the present study, correction coefficients were calculated as the ratio of cumulative estimated mortality to observed mortality throughout the observation period instead for each year, to obtain stable estimates.

We calculated age-specific rates and age-standardized rates (ASRs; standardized to the 1985 model Japanese population, per 100 000 people) of IHBD and EHBD cancer incidence and mortality for all of Japan. The ASR of mortality was calculated for individual years, while the ASR of incidence was calculated for the following 2- or 3-year periods (due to low annual incidence rates): 1985–1986, 1987–1989, 1990–1992, 1993–1995, 1996–1998, 1999–2001, 2002–2004, and 2005–2007. To investigate differences in trends according to age group, we calculated ASRs at all ages (ASR_all_), ASRs for age 30 to 49 years (ASR_30–49_), and ASRs for age 50 years or older (ASR_≥50_).

Long-term trends in ASRs of incidence and mortality were analyzed using a joinpoint regression model.^4^^,^^13^^–^^15^ This model identifies the year in which significant changes in ASR trends occurred, which is called the joinpoint. We set the number of joinpoints to a minimum of 0 and a maximum of 2 (for incidence) or 4 (for mortality) to find the best-fit model using the Monte Carlo permutation method. We also estimated percentage change (PC) in ASR for each segment, which was a 2- or 3-year period for incidence and annually for mortality. A 2-tailed *P* value of less than 0.05 was considered significant. The analysis was performed using Joinpoint software (version 4.0.1) from the Surveillance Research Program of the National Cancer Institute.

## RESULTS

### Incidence

Table 1 shows the cumulative age-specific rates and ASRs of incidence and mortality throughout the observation period. The incidence rates of IHBD cancer for men and women were highest in age groups 80 to 84 and 75 to 79 years, respectively. However, high incidence rates were observed in older age groups in general.

**Table 1.** Cumulative age-specific and age-standardized rates (ASRs) of the incidence (1985–2007) and mortality (1985–2011) of IHBD and EHBD cancer

	Incident				Mortality			
	
	IHBD^a^		EHBD^b^		IHBD		EHBD	
			
	Men	Women	Men	Women	Men	Women	Men	Women
0–4	0.0	0.0	0.0	0.0	0.0	0.0	0.0	0.0
5–9	0.0	0.0	0.0	0.0	0.0	0.0	0.0	0.0
10–14	0.0	0.0	0.0	0.0	0.0	0.0	0.0	0.0
15–19	0.0	0.0	0.0	0.0	0.0	0.0	0.0	0.0
20–24	0.0	0.0	0.0	0.0	0.0	0.0	0.0	0.0
25–29	0.0	0.0	0.0	0.1	0.0	0.0	0.0	0.0
30–34	0.0	0.1	0.2	0.1	0.0	0.0	0.1	0.1
35–39	0.1	0.2	0.5	0.2	0.1	0.1	0.2	0.1
40–44	0.4	0.3	1.0	0.4	0.2	0.1	0.5	0.3
45–49	0.7	0.5	1.6	0.9	0.4	0.2	1.2	0.7
50–54	1.6	0.7	3.3	1.9	0.8	0.4	2.5	1.5
55–59	2.4	1.3	6.7	3.8	1.4	0.8	5.1	2.8
60–64	3.9	1.8	13.0	5.9	2.4	1.3	9.3	4.9
65–69	5.4	2.6	22.5	10.4	3.7	1.9	16.6	8.8
70–74	7.3	3.8	34.1	18.0	5.2	2.9	27.1	14.7
75–79	6.3	5.0	49.1	27.7	7.0	3.9	41.1	23.7
80–84	7.9	4.4	67.1	43.2	8.6	4.9	59.3	35.5
85–	5.9	3.4	82.8	55.9	8.9	5.8	74.6	49.2
ASR_all_^c^	1.2	0.7	5.6	3.1	0.9	0.5	4.4	2.5
ASR_30–49_^d^	0.3	0.2	0.8	0.4	0.2	0.1	0.5	0.3
ASR_≥50_^e^	4.0	2.2	19.8	10.9	3.1	1.7	15.9	9.1

^a^Intrahepatic bile duct.

^b^Extrahepatic bile duct.

^c^Age-standardized rate at all ages.

^d^Age-standardized rate for age 30 to 49.

^e^Age-standardized rate for age 50 years or older.

**Figure 1.**
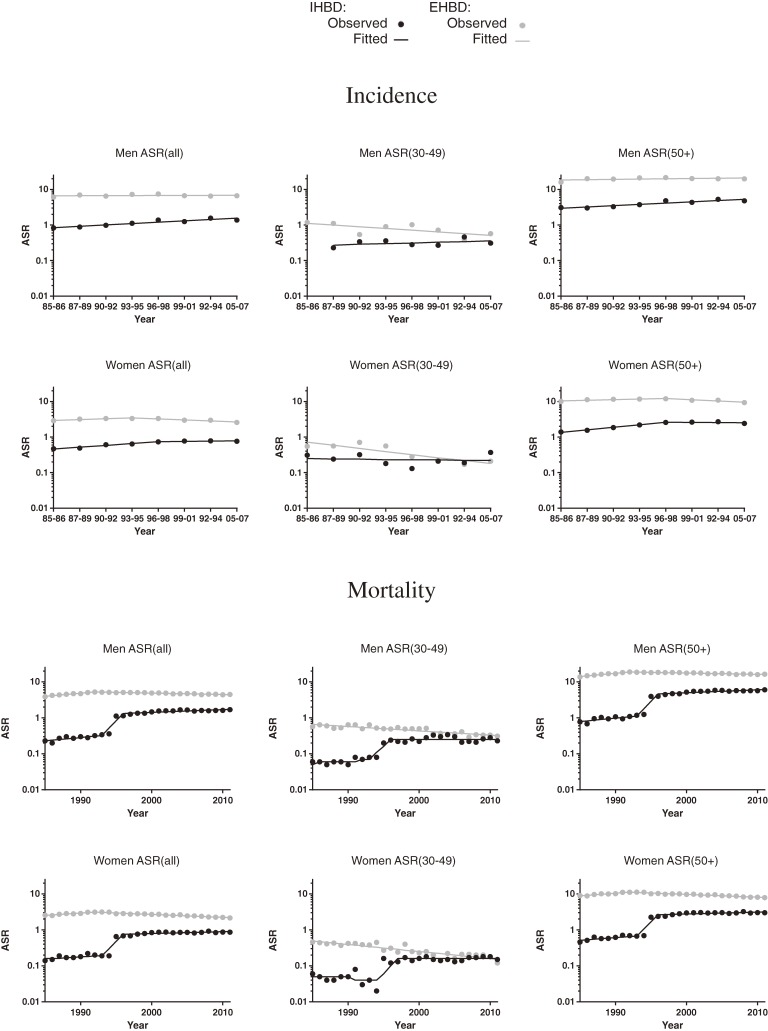
Trends in age-standardized IHBD and EHBD cancer incidence and mortality rates.

Table 2 shows the results of joinpoint regression analysis of the ASRs of incidence and mortality, and Figure 1 shows trends in ASRs of incidence and mortality. The ASR_all_ and ASR_≥50_ of IHBD cancer incidence among men increased by 9.1% and 8.6%, respectively, per 2- or 3-year period throughout the observation period (*P* < 0.05). The values for women also increased by 12.9% and 17.9%, respectively, per 2- or 3-year period until 1996–1998 (*P* < 0.05) and remained stable in recent years. The ASRs_30–49_ of IHBD cancer incidence were stable throughout the observation period in both sexes.

**Table 2.** Results of joinpoint regression analysis of trends in age-standardized incidence (1985–2007) and mortality (1985–2011) rates of IHBD and EHBD cancer

	ASR	Line segment 1					Line segment 2					Line segment 3				
		
		Years		PC^a^	95% CI		Years		PC^a^	95% CI		Years		PC^a^	95% CI	
					
		Start	End		Lower	Upper	Start	End		Lower	Upper	Start	End		Lower	Upper
Incidence																
IHBD																
Men	all	1985–1986	2005–2007	9.1*	5.5	12.8										
	30–49^b^	1987–1989	2005–2007	4.5	−6.2	16.5										
	≥50	1985–1986	2005–2007	8.6*	4.9	12.5										
Women	all	1985–1986	1996–1998	12.9*	6.2	20.0	1996–1998	2005–2007	1.7	−7.7	12					
	30–49	1985–1986	2005–2007	−1.9	−14.7	12.9										
	≥50	1985–1986	1996–1998	17.9*	12.0	24.0	1996–1998	2005–2007	−1.0	−8.6	7.3					
EHBD																
Men	all	1985–1986	2005–2007	0.4	−2.1	3.0										
	30–49	1985–1986	2005–2007	−10.5	−20.3	0.6										
	50–	1985–1986	2005–2007	1.9	−1.3	5.2										
Women	all	1985–1986	1993–1995	5.6	−3.0	15.0	1993–1995	2005–2007	−6.3*	−11.3	−1.1					
	30–49	1985–1986	2005–2007	−18.3*	−26.6	−9.0										
	50–	1985–1986	1995–1998	4.0	−2.9	11.4	1995–1998	2005–2007	−7.8	−17.3	2.8					

Mortality																
IHBD																
Men	all	1985	1993	4.3*	2.0	6.7	1993	1996	60.2*	30.3	97.0	1996	2011	1.8*	0.9	2.7
	30–49	1985	1993	3.1	−2.7	9.1	1993	1996	54.7	−8.6	161.8	1996	2011	0.0	−2.2	2.2
	50–	1985	1993	4.4*	1.9	7.0	1993	1996	60.7*	28.4	101.1	1996	2011	1.9*	0.9	2.9
Women	all	1985	1993	2.7*	0.7	4.8	1993	1996	57.6*	31.2	89.1	1996	2011	1.2*	0.4	2.0
	30–49	1985	1994	−2.6	−7.6	2.6	1994	1997	57.0	−11.8	179.5	1997	2011	0.0	−2.7	2.7
	50–	1985	1993	3.4*	1.4	5.5	1993	1996	57.9*	31.7	89.3	1996	2011	1.2*	0.4	2.0
EHBD																
Men	all	1985	1992	4.1*	3.4	4.8	1992	2011	−0.9*	−1.1	−0.8					
	30–49	1985	2011	−2.8*	−3.3	−2.2										
	50–	1985	1992	4.3*	3.6	5.0	1992	2011	−0.8*	−1.0	−0.7					
Women	all	1985	1992	3.1*	2.1	4.2	1992	2011	−1.9*	−2.1	−1.6					
	30–49	1985	2011	−4.4*	−5.2	−3.5										
	50–	1985	1992	3.4*	2.4	4.5	1992	2011	−1.8*	−2	−1.5					

*Percentage change (PC) significantly different from zero.

^a^Percentage change between 2- or 3-year period for incidence, and annual percentage change for mortality.

^b^Incidence rate was 0 in 1985–86 and was excluded from the analysis.

All ASRs of EHBD cancer incidence among men were stable throughout the observation period. The ASR_all_ of EHBD cancer incidence among women decreased by 6.3% per 3-year period from 1993–1995 (*P* < 0.05), and the ASR_30–49_ decreased by 18.3% per 2- or 3-year period throughout the observation period (*P* < 0.05). The ASR_≥50_ among women was stable throughout the observation period.

The ASRs of EHBD cancer were 1- to 8-fold those of IHBD cancer. However, the differences between the ASRs of IHBD and EHBD cancer decreased over time, because those of IHBD cancer tended to increase or remain stable while those of EHBD cancer tended to decrease or remain stable. In particular, there were only small differences between the ASR_30–49_ of IHBD and EHBD cancer incidence in recent years.

The ASRs of IHBD and EHBD cancer incidence among men were 1- to 2-fold and 2- to 4-fold, respectively, those among women.

### Mortality

High mortality rates were observed in older age groups (Table 1).

Trends in IHBD cancer mortality markedly changed (Table 2 and Figure 1). There were dramatic increases in all ASRs for both sexes in 1995, and APCs between 1993 and 1995 were extremely large (approximately 60% per year). Since 1996, the ASR_all_ and ASR_≥50_ of IHBD cancer mortality increased among men (1.8% and 1.9% per year, respectively) and women (both 1.2% per year) (*P* < 0.05). However, ASR_30–49_ remained stable in both sexes after 1996.

The ASR_all_ and ASR_≥50_ of EHBD cancer increased until 1992 and decreased thereafter in men (−0.9% and −0.8% per year, respectively) and women (−1.9% and −1.8% per year, respectively) (*P* < 0.05). The ASR_30–49_ decreased among men and women by 2.8% and 4.4% per year, respectively, throughout the observation period.

The ASRs of EHBD cancer since 1995 were 1- to 5-fold those of IHBD cancer. The differences between the ASRs of IHBD and EHBD cancer decreased, because the ASRs of IHBD cancer tended to increase or remain stable while those of EHBD cancer tended to decrease since 1992. In particular, there were only small differences between the ASR_30–49_ of IHBD and EHBD cancer in recent years.

The ASRs of IHBD and EHBD cancer among men were 1- to 2-fold those among women.

## DISCUSSION

Using 4 selected population-based cancer registries and vital statistics, we examined IHBD and EHBD cancer incidence and mortality in Japan. Regarding overall ASRs and those in people 50 years or older, IHBD cancer incidence and mortality increased or remained stable in both sexes. EHBD cancer incidence was stable in men and decreased or remained stable in women, while EHBD cancer mortality decreased since 1992 in both sexes. Regarding ASRs in people aged 30 to 49 years, IHBD cancer incidence and mortality were stable, while EHBD cancer incidence and mortality remained stable or decreased throughout the observation period.

A possible explanation for the marked increase in IHBD cancer mortality in 1995 is the adoption of ICD-10 as the classification for causes of death and the simultaneous revision of the death certificate form. The Japanese Ministry of Health, Labour and Welfare reports that these changes affected mortality statistics. In particular, as a result of these changes the liver cirrhosis mortality rate decreased, while liver and IHBD cancer mortality rates have increased since 1995.^16^ The observed marked increases in IHBD cancer mortality since 1995 are consistent with the present results.

**Figure 2.**
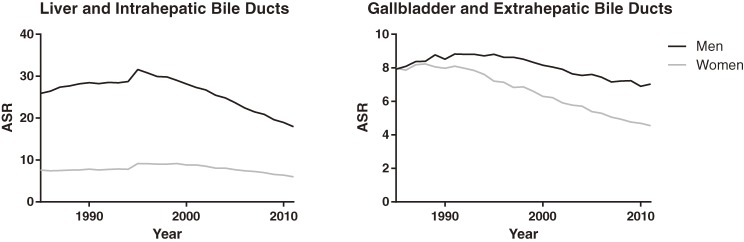
Trends in age-standardized mortality rates.

We calculated provisional ASRs of mortality from “liver and intrahepatic bile ducts” (ICD-9, 155; ICD-10, C22) and “gallbladder and extrahepatic bile ducts” (ICD-9, 156; ICD-10, C23–24). The former increased steeply in 1995, while the latter changed moderately (Figure 2). Thus, our finding of a marked increase only in the ASR of IHBD cancer mortality is reasonable.

IHBD and EHBD cancer incidence rates have increased in the United States.^17^^–^^19^ In England and Wales, incidence rates have increased and decreased, respectively.^20^^,^^21^ Meanwhile, worldwide IHBD and EHBD cancer mortality rates were reported to have increased and decreased, respectively.^18^^,^^22^^–^^24^ Thus, the tendencies observed in Japan in the present study conform to global trends.

However, ASRs reported in the present study differ from those in other countries. Although ASRs of IHBD cancer incidence and mortality in Japan are similar to those in other countries, the ASRs of EHBD cancer in Japan are substantially higher than in other countries. ASRs of IHBD cancer incidence in Japan during 1999–2001 were 1.25 (men) and 0.77 (women), as compared with ASRs of 1.33 (men) and 1.06 (women) in England and Wales.^21^ Furthermore, the ASRs of EHBD cancer incidence in Japan were 6.69 (men) and 2.98 (women), as compared with 0.42 (men) and 0.36 (women) in England and Wales.^21^ It is not clear why the ASR of EHBD cancer incidence is higher in Japan. A US study reported that the ASRs of EHBD cancer differed by ethnicity.^19^ Therefore, genetic and other risk factors may explain the relatively high ASRs in Japan. Future studies should investigate factors related to IHBD and EHBD cancer incidence, to better explain these trends in Japan.

The increasing incidence of IHBD cancer might be due in part to the introduction of advanced imaging modalities such as computed tomography (CT) and endoscopic retrograde cholangiopancreatography (ERCP), although there is no definitive evidence of this.^21^ Another explanation is diagnostic misclassification. Although hilar cholangiocarcinoma (ie, Klatskin tumors) is a cancer of the EHBD, the ICD-O cross-references it with topography codes for either IHBD or EHBD cancer, particularly the ICD-O Second Revision (ICD-O-2). Therefore, hilar cholangiocarcinoma may be mistakenly classified as IHBD cancer,^25^^,^^26^ which may contribute in part to the respective increase and decrease in IHBD and EHBD cancer incidences in England, Wales, and the United States.^27^^,^^28^ However, a previous study concluded that misclassification was not the only cause of increased IHBD cancer incidence.^28^

The changes in trends might be due in part to changes in risk factors. IHBD and EHBD cancer share many risk factors, such as choledochal cysts, cholangitis, inflammatory bowel disease, biliary cirrhosis, cholelithiasis, alcoholic liver disease, nonspecific cirrhosis, diabetes, thyrotoxicosis, chronic pancreatitis, and gallstones.^29^^–^^32^ However, some risk factors are more strongly associated with IHBD than with EHBD cancer, such as obesity, chronic nonalcoholic liver disease, smoking, and hepatitis C virus (HCV) infection.^29^^,^^33^ In particular, HCV-related cirrhosis is a major risk factor for bile duct cancer, especially IHBD cancer.^34^^,^^35^ The prevalence rate of HCV is reported to be high in Japanese born around 1935 (ie, people aged 73–82 years in 2013) and lower in younger Japanese.^36^ This older age group also has a high incidence rate of IHBD cancer. Therefore, the increased rate of IHBD cancer incidence observed in the present study might be affected by both the high rate of HCV infection and older age. If this hypothesis is correct, however, the incidence rate of IHBD cancer should have begun to decrease from the 1990s, along with the incidence rate of liver cancer.^36^ Thus, other risk factors are probably related to the increased incidence of IHBD cancer.

IHBD and EHBD cancers are not well understood, due to confusion regarding their classification and their relative rarity, poor prognosis,^26^ and insufficiently understood risk factors. Therefore, additional studies of the causes of these cancers are required.

Finally, it is important to mention that incidence rates in this study were estimated using data from 4 selected prefectures. There are no data from a nationwide population-based cancer registry in Japan at this time, so the use of tentative data was unavoidable. However, a previous study confirmed the representativeness and homogeneity of these 4 prefectures for all-cancer incidence and mortality.^4^ However, site-specific representativeness and homogeneity were not clear and were not verified for IHBD and EHBD cancer.

In conclusion, since 1992 IHBD cancer incidence and mortality rates overall and among people aged 50 years or older remained stable or increased in Japan, while the EHBD cancer incidence rate remained stable or decreased and the EHBD cancer mortality rate decreased. The incidence and mortality rates of both these cancers remained stable or decreased among people aged 30 to 49 years. These long-term trends in IHBD and EHBD cancer are comparable to those for specific groups, such as workers at printing companies, and are useful for estimating risks of incidence and mortality.

## ONLINE ONLY MATERIALS

Abstract in Japanese.
