# Supplementary material for: Long-term Trends in Incidence and Mortality of Intrahepatic and Extrahepatic Bile Duct Cancer in Japan
Source: J Epidemiol. 2014 May 5;24(3):193–9. doi: 10.2188/jea.JE20130122 (PMC4000766; doi:10.2188/jea.JE20130122)
Supplement: Abstract in Japanese. [file je-24-193-s001.pdf]

日本における肝内胆管がんと肝外胆管がんの罹患率と死亡率の長期トレンド

歌田真依[1]、大野ゆう子[1]、玉木朋子[1]、祖父江友孝[2]、圓藤吟史[3]

[1] 大阪大学大学院医学系研究科数理保健学、[2] 大阪大学大学院医学系研究科社会環境医学、[3] 大阪市立大学大学院医学研究科産業医学

【背景】印刷会社における胆管がんの多発が報告され、関心が高まっている。本研究では、日本における胆管がんの長期トレンドを検討した。

【方法】がん罹患は 1985–2007 年の 4 つの地域がん登録、がん死亡は 1985–2011 年の人口動態統計を用いた。年齢調整率は Joinpoint 回帰モデルを用いて解析した。

【結果】全年齢の肝内胆管がんの罹患率は、男性では観察期間中増加し、女性では 1996–1998 年まで増加し、それ以降は一定であった。肝外胆管がんの罹患率は、男性では一定で、女性では 1993–1995 年から減少した。30–49 歳の肝内胆管と肝外胆管の罹患率は、一定または減少した。男女共に肝内胆管がんの死亡率は 1996 年から増加し、肝外胆管の死亡率は 1992 年から減少した。30–49 歳の肝内胆管の死亡率は一定で、肝外胆管の死亡率は減少した。

【結論】肝内胆管がんの罹患率と死亡率は、観察期間中一定か増加していた。一方、肝外胆管がんの罹患率は一定か減少で、死亡率は 1992 年から減少していた。30–49 歳では、肝内胆管と肝外胆管の罹患率と死亡率は一定か減少していた。

キーワード：肝内胆管がん、肝外胆管がん、罹患、死亡
